# Diagnostic value of triglyceride-rich lipoprotein cholesterol for coronary artery lesions and its predictive significance for long-term prognosis in elderly patients with coronary heart disease

**DOI:** 10.3389/fcvm.2025.1638134

**Published:** 2026-01-08

**Authors:** Wei Qi, Fei Jiang, She-Bao Bai, Yazheng Zhang, Zhi-Hui Fang, Yu-Si Zhou, Rui Chen, Zong-Hao Zhang, Lin Wang, Wenyu Li, Ao Wei, Tingting Li, Jiachun Lang, Kai Hou, Haihui Yang, Hongliang Cong

**Affiliations:** 1 Department of Cardiology, Tianjin Chest Hospital, Tianjin, China; 2 Department of Cardiology, Chest Hospital, Tianjin University, Tianjin, China; 3 Research Center, Pu’er People’s Hospital, Yunnan, China; 4 School of Medicine, The Affiliated Pu'er Hospital of Kunming University of Science and Technology, Kunming University of Science and Technology, Yunnan, China; 5 Department of Radiology, Pu’er People’s Hospital, Yunnan, China; 6 Department of Cardiology, Pu’er People’s Hospital, Yunnan, China

**Keywords:** coronary artery disease, diagnostic model, dyslipidemia, non-traditional lipid indicator, prediction model, triglyceride-rich lipoprotein cholesterol

## Abstract

**Objective:**

To assess the diagnostic and prognostic value of triglyceride-rich lipoprotein cholesterol (TRL-C) in patients with coronary artery disease in the elderly.

**Methods:**

This study enrolled 220 patients with coronary artery disease in the elderly undergoing coronary angiography at Tianjin Chest Hospital (January–December 2020) with a four-year follow-up. Coronary lesions were classified as mild (SYNTAX score 1–22) or moderate-to-severe (score ≥23). Logistic regression analyzed TRL-C's association with lesion severity, while Cox regression evaluated its predictive capacity for all-cause mortality.

**Results:**

For coronary lesion severity prediction, TRL-C-based models achieved AUCs of 0.76 (95% CI: 0.68–0.85) and 0.77 (95% CI: 0.64–0.90) in training and validation sets, respectively, with decision curve analysis (DCA) confirming clinical utility. For all-cause mortality prediction, models demonstrated higher discriminative performance, with training and validation AUCs of 0.94 (95% CI: 0.89–0.99) and 0.92 (95% CI: 0.86–0.99), respectively, supported by favorable DCA results.

**Conclusion:**

TRL-C strongly predicts both coronary lesion severity and long-term mortality in patients with coronary artery disease in the elderly, offering a potential biomarker for risk stratification and personalized management.

## 1 Introduction

Dyslipidemia is an important risk factor for cardiovascular diseases, particularly the elevated levels of serum low-density lipoprotein cholesterol (LDL-C), which are key factors in the occurrence and development of atherosclerotic lesions (1). As a major cause of morbidity and mortality among the elderly globally, the long-term prognosis assessment of cardiovascular diseases remains a critical aspect of clinical decision-making (2). Although LDL-C remains a central lipid target, its limitations in capturing residual cardiovascular risk have become evident, highlighting the emerging role of triglyceride-rich lipoprotein cholesterol (TRL-C) as a complementary indicator of remnant lipoprotein atherogenicity (3–5). In recent years, a series of studies from European, American, and Asian countries have indicated that novel non-traditional lipid indicators, particularly TRL-C, are closely associated with the occurrence and progression of atherosclerotic lesions (4–7).

TRL-C includes chylomicron remnants, very low-density lipoprotein (VLDL), and intermediate-density lipoprotein (IDL) in a non-fasting state, as well as VLDL and IDL in a fasting state. TRL-C can be calculated using the formula “Total Cholesterol (TC)—High-density lipoprotein cholesterol (HDL-C)—LDL-C” (6, 7). Although evidence of the correlation between TRL-C and cardiovascular diseases is increasing, most of these studies are based on database analyses or research focused on Caucasian populations in Europe and America (8–10). Currently, research on the correlation between TRL-C and cardiovascular diseases, especially coronary heart disease, in the elderly population in China is still insufficient. Due to age-related metabolic changes, dyslipidemia, and the high prevalence of comorbidities, as well as shifts in diet and lifestyle associated with urbanization, elderly population in China exhibit greater susceptibility. Therefore, this study was designed to test the hypothesis that higher levels of TRL-C are associated with greater severity of coronary artery lesions and have diagnostic value for identifying moderate-to-severe lesions in the elderly population. Furthermore, we hypothesized that TRL-C acts as an independent predictor of four-year all-cause mortality and that incorporating TRL-C into multivariate Cox regression models could enhance risk stratification and support individualized prognostic assessment.

## 2 Methods

### 2.1 Patients

220 elderly angiography patients with coronary heart disease who underwent Coronary (CAG) examination at Tianjin Chest Hospital between January 1, 2020, and December 31, 2020, were selected. Data on patients' gender, age, height, weight, blood pressure, heart rate, and other general information, as well as routine laboratory tests including blood routine, liver function, kidney function, electrolytes, blood lipids, fasting blood glucose, coagulation function, troponin T, amino-terminal pro-brain natriuretic peptide, and high-sensitivity C-reactive protein, were collected. The SYNTAX score was calculated by logging onto the website (http://www.syntaxscore.com), and patients were grouped based on their scores: mild lesion group (1–22 points) and moderate to severe lesion group (23 points and above). The SYNTAX score cutoff of 23 was selected according to the standard classification from the SYNTAX trial, which differentiates low (≤22), intermediate (23–32), and high (≥33) anatomical complexity. This threshold reflects significant differences in lesion complexity, procedural risk, and long-term cardiovascular outcomes, and has been widely adopted in both clinical practice and research for stratifying revascularization strategies and prognosis. All patients were followed up for four years. Clinical follow-up data over four years were collected through outpatient visits and telephone interviews. All patients signed informed consent documents upon admission, including a statement for the use of medical records for research purposes. Inclusion criteria: ① All cases met the 2023 AHA “Guideline for the Management of Patients with Chronic Coronary Artery Disease” or the 2019 Chinese Medical Association Emergency Physician Branch “Guideline for Rapid Diagnosis and Treatment of Acute Coronary Syndrome”; ② All cases underwent coronary angiography in our hospital showing at least one major coronary artery with stenosis ≥50%; ③ Complete clinical data; ④ Voluntary participation in this study. Exclusion criteria: ① Severe heart failure [left ventricular ejection fraction (LVEF) < 30%] or non-ischemic cardiomyopathy, valvular heart disease; ② Acute cerebrovascular or peripheral arterial disease; ③ Severe liver and kidney dysfunction; ④ Thyroid disease; ⑤ Malignant tumors; ⑥ Acute or chronic infections or inflammation. All eligible patients who met the inclusion and exclusion criteria were consecutively enrolled during the study period to minimize potential selection bias.

### 2.2 Statistical methods

This study used R (4.4.1), Zstats v1.0 (https://www.zstats.net), and SPSS 22.0 for data processing and analysis. For variables that conform to a normal distribution, independent sample *t*-tests were used, and results were expressed as “mean ± standard deviation”; for variables that do not conform to a normal distribution, non-parametric independent sample tests were used, and results were described as “median (interquartile range)”. Normality was assessed using the Shapiro–Wilk test, which is appropriate for small sample sizes. We used the random sampling principle in simple cross-validation to divide the overall dataset into training and validation sets in a 7:3 ratio. This method has been widely adopted in the construction of clinical prediction models (11, 12). Specifically, pseudo-random number sequences are generated through the R language, and a random seed is set to ensure that the partitioning process can be strictly reproduced. This method is widely accepted in clinical predictive model research and can effectively support the reliability of model validation.

Univariate and multivariate logistic regression analyses were conducted to determine risk factors in the cohort. Variables with *P* values <0.05 in multivariate regression analysis were used to form a nomogram. Receiver operating characteristic (ROC) curves were used to assess the discriminative ability of the nomogram. The area under the curve (AUC) indicates accuracy, with larger AUC values indicating higher accuracy. AUC values range from 0.5 to 1.0, where 0.5 indicates random chance and 1.0 indicates perfect compliance. An AUC value greater than 0.7 indicates a reasonable estimate. Decision curve analysis (DCA) was used to assess the clinical utility for all patients and quantify net benefits at different threshold probabilities. A two-tailed *P* value <0.05 was considered statistically significant.

## 3 Results

### 3.1 Clinical and demographic information of enrolled patients

**Figure 1:**
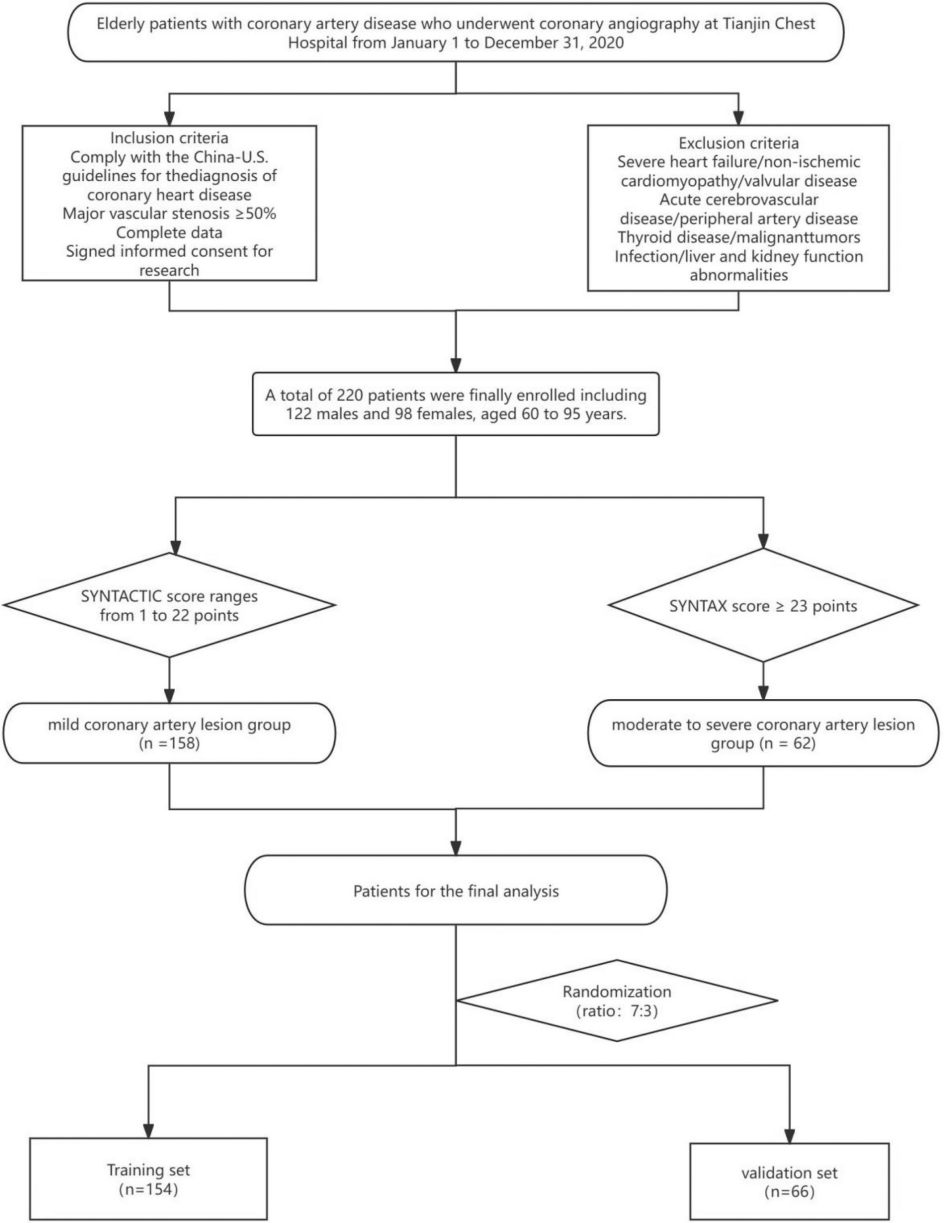
Patients enrollment flowchart. Flowchart of patient enrollment and grouping: This diagram outlines the selection process for elderly patients with coronary artery disease (undergoing coronary angiography at Tianjin Chest Hospital between January 1 and December 31, 2020). Patients were included based on diagnostic guidelines, complete data, and informed consent, while those with severe comorbidities (e.g., heart failure, acute cerebrovascular disease) were excluded. A total of 220 eligible patients (122 males, 98 females; aged 60–93 years) were stratified into a mild coronary artery lesion group (SYNTAX score: 1–22 points, *n* = 138) and a moderate-to-severe lesion group (SYNTAX score ≥23 points, *n* = 62). These patients were further randomized (ratio 7:3) into a training set (*n* = 154) and validation set (*n* = 66) for subsequent analysis.

A total of 220 elderly patients with coronary heart disease who underwent CAG examination at Tianjin Chest Hospital from January 1, 2020, to December 31, 2020, were included and randomly divided into two cohorts (7:3), including a training cohort (*n* = 154) and a validation cohort (*n* = 66). The patient selection process is shown in (Figure 1), and the clinical and demographic information of the enrolled patients is presented in (Table 1).

**Table 1:** Clinical and demographic information.

Variables	Total (*n* = 220)	Mild coronary artery disease group (*n* = 158)	Moderate to severe coronary artery disease group (*n* = 62)	Statistic	*P*
Age, M (Q₁, Q₃)	66.00 (63.00, 69.00)	66.00 (63.00, 69.00)	66.00 (63.00, 71.00)	Z = −1.51	0.132
ALB, M (Q₁, Q₃), g/L	41.60 (39.45, 43.90)	41.95 (39.70, 44.30)	41.30 (39.20, 42.80)	Z = −1.82	0.069
LDL-C, M (Q₁, Q₃), mmol/L	2.46 (1.97, 3.31)	2.52 (2.03, 3.33)	2.42 (1.85, 3.26)	Z = −0.71	0.477
HDL-C, M (Q₁, Q₃), mmol/L	1.38 (0.92, 1.99)	1.47 (0.99, 2.04)	1.17 (0.78, 1.70)	Z = −2.62	0.009
TG, M (Q₁, Q₃), mmol/L	1.11 (0.86, 1.50)	1.08 (0.88, 1.36)	1.29 (0.80, 4.20)	Z = −1.89	0.059
TC, M (Q₁, Q₃), mmol/L	4.09 (3.48, 5.08)	4.03 (3.43, 4.96)	4.33 (3.51, 5.40)	Z = −1.29	0.197
GGT, M (Q₁, Q₃), U/L	22.85 (16.38, 36.48)	22.10 (16.02, 35.22)	25.50 (17.82, 40.58)	Z = −1.46	0.144
Cr, M (Q₁, Q₃), μmol/L	73.00 (61.00, 85.00)	70.00 (61.00, 83.00)	80.00 (67.00, 89.00)	Z = −2.65	0.008
UA, M (Q₁, Q₃), μmol/L	313.50 (254.00, 377.25)	299.00 (253.00, 377.00)	342.00 (267.00, 377.50)	Z = −1.40	0.163
K, Mean ± SD, mmol/L	4.00 ± 0.41	4.00 ± 0.41	4.00 ± 0.41	t = 0.08	0.934
Glu, M (Q₁, Q₃), mmol/L	5.82 (5.09, 6.96)	5.68 (5.00, 6.68)	6.36 (5.38, 7.46)	Z = −2.20	0.028
Hs-CRP, M (Q₁, Q₃)	1.56 (0.70, 4.00)	1.52 (0.70, 3.92)	1.82 (0.72, 4.29)	Z = −0.29	0.770
Lp(a), M (Q₁, Q₃), mmol/L	28.75 (12.30, 80.48)	30.45 (14.10, 86.72)	19.70 (8.48, 73.67)	Z = −1.69	0.090
ApoA, M (Q₁, Q₃), g/L	1.25 (1.03, 1.53)	1.29 (1.07, 1.56)	1.14 (0.98, 1.40)	Z = −2.47	0.014
ApoB, M (Q₁, Q₃), g/L	0.94 (0.79, 1.16)	0.92 (0.78, 1.15)	0.98 (0.81, 1.18)	Z = −0.86	0.388
cTnT, M (Q₁, Q₃), μg/L	0.01 (0.01, 0.08)	0.01 (0.01, 0.03)	0.03 (0.01, 0.26)	Z = −3.75	<.001
NTproBNP, M (Q₁, Q₃), ng/L	137.60 (57.43, 514.05)	113.30 (57.11, 379.00)	223.00 (57.96, 640.80)	Z = −1.38	0.167
WBC, M (Q₁, Q₃), ×10^9^/L	6.94 (5.95, 8.26)	6.79 (5.87, 7.91)	7.44 (5.97, 8.39)	Z = −1.64	0.102
NEUT%, M (Q₁, Q₃), %	64.50 (58.65, 71.47)	63.70 (58.30, 70.60)	66.70 (59.74, 72.90)	Z = −1.51	0.131
LY%, Mean ± SD, %	27.44 ± 8.60	27.77 ± 8.47	26.63 ± 8.94	t = 0.88	0.382
MON%, Mean ± SD, %	6.11 ± 1.70	6.08 ± 1.65	6.19 ± 1.82	t = −0.45	0.656
NEUT, M (Q₁, Q₃), ×10^9^/L	4.47 (3.62, 6.09)	4.34 (3.55, 5.66)	5.41 (3.92, 6.53)	Z = −2.55	0.011
LYMPH, M (Q₁, Q₃), ×109/L	1.85 (1.45, 2.33)	1.89 (1.46, 2.27)	1.73 (1.43, 2.48)	Z = −0.01	0.994
MONO, M (Q₁, Q₃), ×10^9^/L	0.42 (0.32, 0.53)	0.39 (0.31, 0.52)	0.47 (0.34, 0.53)	Z = −1.60	0.109
BASO, M (Q₁, Q₃), ×10^9^/L	0.10 (0.06, 0.17)	0.10 (0.06, 0.16)	0.11 (0.06, 0.18)	Z = −0.96	0.337
RBC, M (Q₁, Q₃), ×10^12^/L	4.43 (4.12, 4.79)	4.42 (4.12, 4.78)	4.43 (4.12, 4.79)	Z = −0.39	0.693
Hb, Mean ± SD, g/L	138.08 ± 16.42	138.62 ± 15.14	136.74 ± 19.35	t = 0.76	0.450
PLT, M (Q₁, Q₃), ×10^9^/L	231.50 (197.00, 266.75)	236.00 (198.00, 273.00)	224.00 (185.00, 255.00)	Z = −1.84	0.066
PT, M (Q₁, Q₃), s	13.10 (12.70, 13.50)	13.10 (12.70, 13.50)	13.00 (12.70, 13.60)	Z = −0.35	0.724
INR, M (Q₁, Q₃)	1.00 (0.96, 1.04)	1.00 (0.96, 1.04)	1.00 (0.96, 1.05)	Z = −0.29	0.770
APTT, M (Q₁, Q₃), s	35.80 (33.00, 38.55)	35.65 (32.82, 38.30)	36.60 (34.30, 40.40)	Z = −1.80	0.072
fib, M (Q₁, Q₃), g/L	3.11 (2.70, 3.54)	3.10 (2.68, 3.58)	3.15 (2.72, 3.49)	Z = −0.20	0.841
TT, M (Q₁, Q₃), s	17.40 (16.70, 18.20)	17.30 (16.70, 18.28)	17.60 (16.78, 18.02)	Z = −0.35	0.724
Dimer, M (Q₁, Q₃), mg/L	0.29 (0.22, 0.43)	0.30 (0.22, 0.43)	0.29 (0.22, 0.42)	Z = −0.08	0.940
BUN, M (Q₁, Q₃), mmol/L	5.30 (4.50, 6.30)	5.20 (4.30, 6.20)	5.40 (4.80, 6.35)	Z = −1.60	0.110
Hcy, M (Q₁, Q₃), μmol/L	13.70 (11.40, 17.90)	13.65 (11.40, 17.30)	14.90 (11.45, 18.30)	Z = −0.67	0.500
T3, M (Q₁, Q₃), nmol/L	1.50 (1.33, 1.69)	1.50 (1.34, 1.74)	1.48 (1.30, 1.62)	Z = −0.72	0.472
T4, M (Q₁, Q₃), nmol/L	106.35 (94.89, 122.47)	108.40 (95.43, 123.95)	104.40 (90.36, 120.05)	Z = −0.93	0.353
TSH, M (Q₁, Q₃), mIU/L	1.79 (1.23, 2.61)	1.86 (1.25, 2.55)	1.71 (1.19, 2.66)	Z = −0.29	0.769
Asp, M (Q₁, Q₃), U/L	19.80 (16.33, 27.50)	19.70 (16.40, 27.00)	20.90 (16.20, 34.40)	Z = −0.87	0.382
CK, M (Q₁, Q₃), U/L	99.00 (64.25, 209.25)	98.00 (64.00, 161.00)	121.00 (69.00, 389.00)	Z = −1.91	0.056
CKMB, M (Q₁, Q₃), U/L	17.00 (14.00, 26.00)	17.00 (13.00, 22.00)	19.00 (15.00, 39.00)	Z = −1.95	0.051
LDH, M (Q₁, Q₃), U/L	188.00 (162.00, 238.50)	183.00 (161.00, 219.00)	211.00 (164.00, 266.00)	Z = −2.27	0.023
HBDH, M (Q₁, Q₃), U/L	147.50 (130.00, 193.00)	146.00 (129.00, 181.00)	166.00 (136.00, 228.00)	Z = −1.89	0.059
LA, M (Q₁, Q₃), mm	38.00 (35.00, 41.00)	38.00 (35.00, 41.00)	39.00 (36.00, 40.75)	Z = −1.21	0.226
LVE, M (Q₁, Q₃), mm	51.00 (48.00, 54.00)	50.00 (48.00, 53.00)	52.00 (48.00, 56.00)	Z = −1.38	0.167
RA, M (Q₁, Q₃), mm	35.50 (33.00, 37.00)	35.00 (33.00, 37.00)	36.00 (34.00, 38.00)	Z = −1.21	0.226
RV, M (Q₁, Q₃), mm	17.00 (16.00, 18.00)	17.00 (16.00, 18.00)	16.50 (16.00, 17.00)	Z = −1.00	0.316
LVEF, M (Q₁, Q₃), %	60.00 (56.00, 63.00)	61.00 (58.00, 63.00)	58.00 (52.25, 63.00)	Z = −2.21	0.027
PAP, M (Q₁, Q₃), mmHg	30.00 (30.00, 30.00)	30.00 (30.00, 30.00)	30.00 (30.00, 30.00)	Z = −0.86	0.388
TRL-C, M (Q₁, Q₃), mmol/L	−0.35 (−0.55, −0.06)	−0.39 (−0.62, −0.16)	−0.10 (−0.43, 0.09)	Z = −4.94	<.001
Sex, n(%)				*χ*^2^ = 2.87	0.090
Female	122 (55.45)	82 (51.90)	40 (64.52)		
Male	98 (44.55)	76 (48.10)	22 (35.48)		

Data are presented as median (first quartile, third quartile) [M (Q₁, Q₃)] for non-normally distributed continuous variables, mean ± standard deviation (Mean ± SD) for normally distributed continuous variables, and *n* (%) for categorical variables. Statistical analyses were performed using the Mann–Whitney *U*-test (Z value) for non-normally distributed continuous variables, independent samples *t*-test (*t* value) for normally distributed continuous variables, and Chi-square test (χ^2^ value) for categorical variables. A two-tailed *P* value < 0.05 was considered statistically significant. *t*, *t*-test; Z, Mann–Whitney test; χ^2^, Chi-square test.

### 3.2 Construction of a prediction model for the degree of coronary artery lesions in elderly patients with coronary heart disease

Based on the results of univariate logistic regression analysis (Table 2), all significant variables in the univariate analysis, including HDL-C, triglycerides (TG), activated partial thromboplastin time (APTT), thrombin time (TT), and TRL-C, were included in the multivariate logistic regression analysis (Table 3). The results indicated that high-density lipoprotein cholesterol and TRL-C are independent factors for predicting the severity of coronary lesions.

**Table 2:** Results of univariate logistic regression.

Variables	S.E	Z	*P*	OR (95% CI)
Age	0.03	0.99	0.323	1.03 (0.97–1.10)
ALB	0.06	−1.28	0.202	0.93 (0.83–1.04)
LDL-C	0.18	−0.24	0.810	0.96 (0.67–1.37)
HDL-C	0.26	−2.10	0.035	0.57 (0.34–0.96)
TG	0.13	3.85	<0.001	1.67 (1.29–2.16)
TC	0.15	1.30	0.194	1.22 (0.90–1.64)
GGT	0.01	−0.14	0.886	1.00 (0.99–1.01)
Cr	0.01	1.09	0.276	1.01 (0.99–1.03)
UA	0.00	0.37	0.710	1.00 (1.00–1.00)
K	0.42	0.11	0.914	1.05 (0.46–2.40)
Glu	0.07	1.75	0.080	1.13 (0.99–1.30)
hs-CRP	0.02	−0.73	0.467	0.98 (0.94–1.03)
Lp(a)	0.00	−1.15	0.251	1.00 (0.99–1.00)
ApoA	0.49	−1.70	0.089	0.43 (0.17–1.14)
ApoB	0.59	0.80	0.425	1.60 (0.50–5.10)
cTnT	0.20	1.28	0.201	1.29 (0.87–1.89)
NTproBNP	0.00	0.80	0.423	1.00 (1.00–1.00)
WBC	0.09	1.57	0.117	1.15 (0.97–1.36)
NEUT%	0.02	1.17	0.241	1.02 (0.99–1.06)
LY%	0.02	−1.33	0.182	0.97 (0.93–1.01)
MON%	0.10	0.44	0.658	1.05 (0.85–1.28)
NEUT	0.06	1.55	0.122	1.10 (0.97–1.25)
LYMPH	0.26	−0.49	0.626	0.88 (0.53–1.46)
MONO	0.96	0.89	0.374	2.35 (0.36–15.40)
BASO	1.95	0.73	0.467	4.14 (0.09–190.51)
RBC	0.37	−0.30	0.765	0.89 (0.43–1.86)
Hb	0.01	−0.61	0.545	0.99 (0.97–1.02)
PLT	0.00	−1.29	0.197	1.00 (0.99–1.00)
PT	0.12	1.05	0.295	1.14 (0.89–1.45)
INR	1.01	0.85	0.394	2.36 (0.33–16.90)
APTT	0.02	1.97	0.048	1.04 (1.01–1.07)
FIB	0.26	−1.32	0.187	0.71 (0.43–1.18)
TT	0.00	2.09	0.037	1.01 (1.01–1.02)
D-Dimer	0.40	−0.07	0.942	0.97 (0.45–2.11)
BUN	0.09	1.17	0.241	1.11 (0.93–1.33)
Hcy	0.02	1.22	0.222	1.02 (0.99–1.05)
T3	1.11	−1.16	0.248	0.28 (0.03–2.43)
T4	0.01	−0.03	0.977	1.00 (0.97–1.03)
TSH	0.08	1.14	0.252	1.10 (0.94–1.28)
Asp	0.01	1.76	0.078	1.01 (1.00–1.03)
CK	0.00	1.67	0.094	1.00 (1.00–1.00)
CKMB	0.00	1.94	0.052	1.00 (1.00–1.01)
LDH	0.00	0.70	0.481	1.00 (1.00–1.00)
HBDH	0.00	0.70	0.482	1.00 (1.00–1.00)
LA	0.03	0.45	0.651	1.01 (0.96–1.07)
LVE	0.04	0.65	0.514	1.02 (0.95–1.10)
RA	0.05	0.72	0.472	1.04 (0.94–1.14)
RV	0.10	−0.29	0.771	0.97 (0.79–1.19)
LVEF	0.02	−1.37	0.171	0.97 (0.93–1.01)
PAP	0.04	0.78	0.433	1.03 (0.96–1.11)
TRL-C(lg)	0.57	3.72	<0.001	8.48 (2.75–26.16)

This table presents the results of Single-factor logistic regression analysis to explore independent clinical, laboratory, and echocardiographic factors associated with moderate-to-severe coronary artery disease (CAD). The dependent variable was CAD severity, dichotomized as moderate-to-severe CAD (case group) vs. mild CAD (reference group). The independent variables included demographic characteristics (age, sex was not included as it was not listed in the table), serum biochemical indicators, lipid profiles, inflammatory markers, cardiac biomarkers, hematological parameters, coagulation function indices, thyroid function indicators, and echocardiographic measurements. Statistical indicators are defined as follows: S.E: Standard error of the regression coefficient; Z: Z-statistic for testing the significance of the regression coefficient; *P*: *P* value (two-tailed, *P* < 0.05 was considered statistically significant); OR (95% CI): Odds ratio with 95% confidence interval, representing the relative risk of developing moderate-to-severe coronary artery disease associated with a unit change in the independent variable.

**Table 3:** Results of multifactorial logistic regression.

Variables	S.E	Z	*P*	OR (95% CI)
HDL-C	0.24	−2.34	0.019	0.57 (0.36–0.91)
TRL-C(lg)	0.63	4.04	<0.001	12.71 (3.71–43.58)

This table presents the results of multivariate logistic regression analysis to identify independent factors associated with moderate-to-severe coronary artery disease (reference group: mild coronary artery disease). The dependent variable was the severity of coronary artery disease (dichotomized as mild vs. moderate-to-severe), and the independent variables included high-density lipoprotein cholesterol (HDL-C) and log-transformed triglyceride-cholesterol ratio [TRL-C(lg)]. Statistical indicators are defined as follows: S.E: Standard error of the regression coefficient; Z: Z-statistic for testing the significance of the regression coefficient; *P*: *P* value (two-tailed, *P* < 0.05 was considered statistically significant); OR (95% CI): Odds ratio with 95% confidence interval, representing the relative risk of developing moderate-to-severe coronary artery disease associated with a unit change in the independent variable.

The OR value for TRL-C was 12.71 (95% confidence interval, 95% CI: 3.71–43.58), *P* < 0.001, which is statistically significant. TRL-C is a risk factor for moderate to severe coronary lesions (Table 3). The OR value for HDL-C was 0.57 (95% CI: 0.36–0.91), which is statistically significant.Relatively, HDL-C is a protective factor against coronary lesions (Table 3).

**Figure 2:**
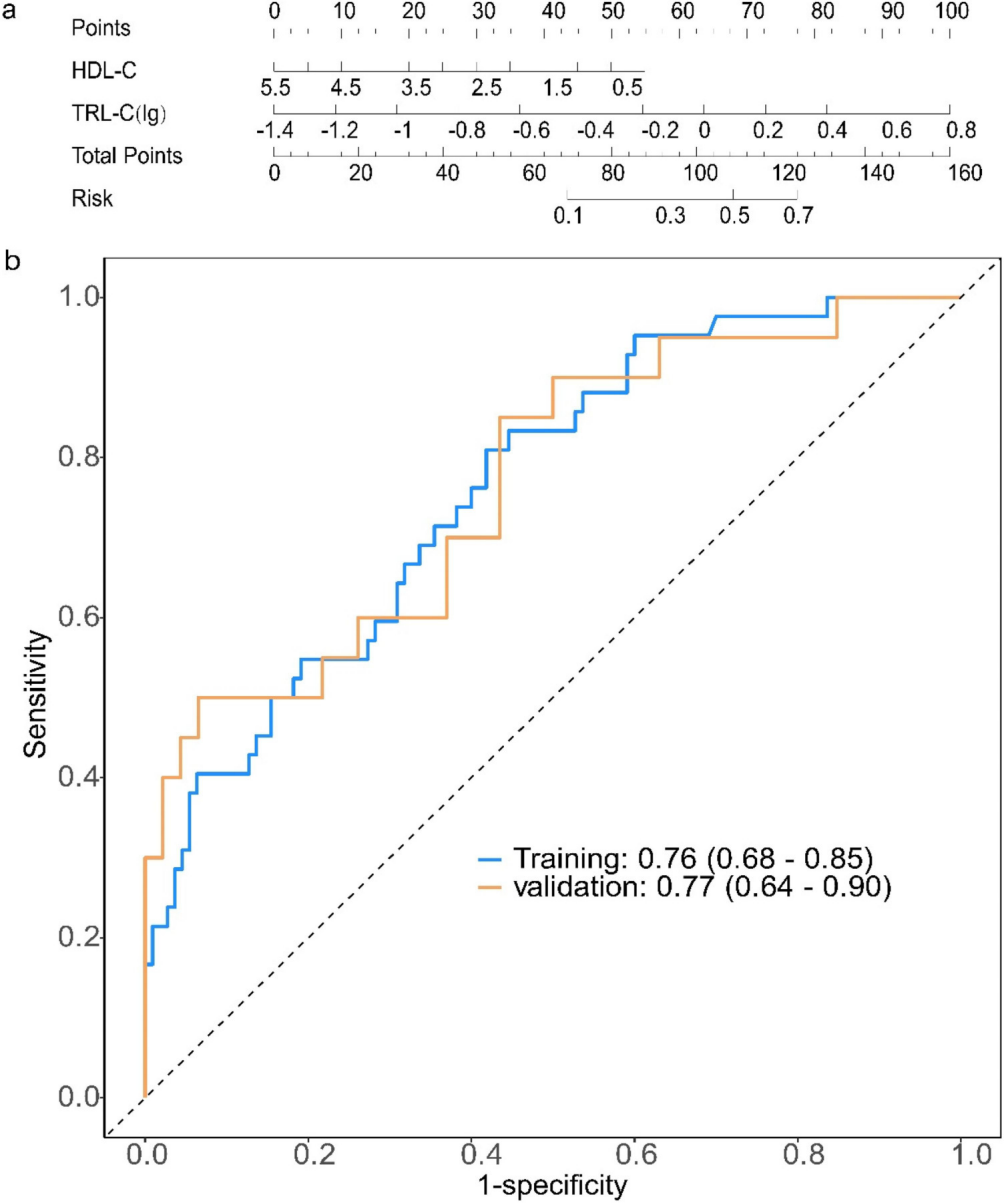
Construction and evaluation of a prediction model for the severity of coronary artery lesions in elderly patients with coronary heart disease. **(a)** Establishment of a nomogram prediction model for the training cohort; Clinical and biochemical variables [HDL-C, high-density lipoprotein cholesterol; TRL-C(lg), log-transformed triglyceride-rich lipoprotein cholesterol] are assigned corresponding points (top axis). The total points (sum of individual variable points) map to the predicted risk (right axis), enabling quantitative risk assessment for the target outcome. Each variable is scored on a scale of 0 to 100, and then the scores for each variable are summed. This total is positioned on the total score axis, allowing us to predict the probability of coronary artery lesion severity. For HDL-C, the scale range is 0.5–5.5. For TRL-C, the variable takes the logarithmic value, with a scale range of −1.4–0.8. **(b)** Receiver operating characteristic (ROC) curves for the training set and validation set. AUC, the area under the receiver-operating characteristic. ROC curves of the model in training (blue) and validation (orange) cohorts: The area under the curve (AUC, 95% confidence interval) is 0.76 (0.68–0.85) in the training cohort and 0.77 (0.64–0.90) in the validation cohort, indicating consistent discriminative ability of the model across datasets.

Using the predictive factors from the multivariate logistic regression of the training cohort, a nomogram prediction model was established (Figure 2a). The total score can be calculated by summing the points for each variable, indicating the probability of each patient developing moderate to severe coronary lesions.

### 3.3 Evaluation of a prediction model for the severity of coronary artery lesions in elderly patients with coronary heart disease

For the coronary lesion severity prediction model, the ROC curve showed that the AUC for the training and validation sets was 0.76 (95% CI: 0.68–0.85) and 0.77 (95% CI: 0.64–0.90) (Figure 2b). We have added the calibration curve of the logistic regression model to further evaluate the model's predictive accuracy (Supplementary Figure S1).

**Figure 3:**
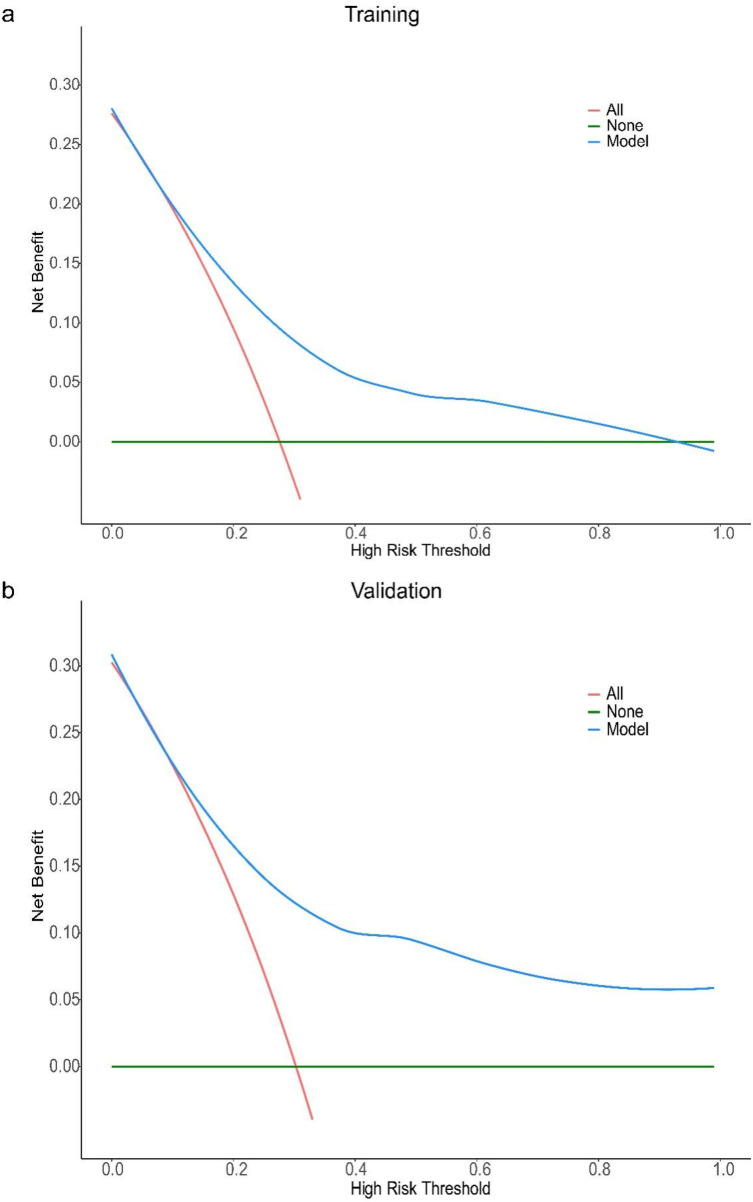
DCA curve of the prediction model for the severity of coronary artery lesions in elderly patients with coronary heart disease. Decision curve analysis (DCA) for the model in training **(a)** and validation **(b)** cohorts. The *x*-axis represents the high-risk threshold (probability of the event), and the *y*-axis represents the net benefit. The “Model” curve (blue) demonstrates the net benefit of using the proposed model to guide decision-making; the “All” curve (red) represents the scenario where all individuals are classified as high-risk; the “None” curve (green) represents the scenario where no individuals are classified as high-risk. In both cohorts, the proposed model (blue curve) maintains a higher net benefit across most thresholds (vs. “All” and “None”), indicating favorable clinical utility.

The results of Decision curve analysis (DCA) show (Figures 3a,b): In both the training cohort and the validation cohort, the “Model” curve corresponding to this model consistently remains above the “All” (all determined high risk) and “None” (all not determined high risk) curves across all high-risk threshold ranges (0–1.0), indicating that the model can provide a net clinical benefit superior to “extreme strategies” by balancing true positive gains and false positive risks at any clinical decision threshold.

It should be noted that in the training cohort, when the high-risk threshold > 0.4, the model's net benefit shows a slight decline — this is due to the mathematical properties of DCA: an increase in the high-risk threshold reduces the number of samples classified as “high risk”, leading to a decrease in the absolute number of true positive cases, thus resulting in a natural mild fluctuation in net benefit. However, in this scenario, the model's net benefit remains positive and significantly better than the “All/None” strategy. From a clinical practice perspective, a high-risk threshold > 0.4 is rarely used in disease risk stratification (commonly used thresholds are often 0.1–0.3), and the trend of the model's net benefit in the validation cohort is consistent with that in the training cohort, further supporting the stability and generalizability of the results. In summary, this model demonstrates good clinical practicality.

### 3.4 Construction of the all-cause mortality prediction model for elderly patients with coronary heart disease

According to the results of the univariate COX regression analysis (Table 4), all significant variables in the univariate analysis, including TG, TC, gamma-glutamyl transferase (GGT), uric acid (UA), high-sensitivity C-reactive protein (hs-CRP), apolipoprotein B (ApoB), neutrophil count (NEUT), creatine kinase (CK), lactate dehydrogenase (LDH), α-Hydroxybutyrate dehydrogenase (HBDH), left ventricular ejection fraction (LVEF), pulmonary arterial pressure (PAP), and TRL-C, were included in the multivariate logistic regression analysis (Table 5). The results indicate that GGT, LVEF, and TRL-C are independent factors predicting all-cause mortality in elderly patients with coronary heart disease.

**Table 4:** Results of univariate COX regression.

Variables	S.E	Z	*P*	HR (95% CI)
Age	0.06	0.90	0.368	1.06 (0.94–1.19)
ALB	0.08	−1.02	0.306	0.92 (0.79–1.08)
LDL-C	0.31	0.95	0.344	1.34 (0.73–2.47)
HDL-C	0.71	−1.76	0.078	0.28 (0.07–1.15)
TG	0.08	3.79	<0.001	1.34 (1.15–1.55)
TC	0.23	2.24	0.025	1.67 (1.07–2.63)
GGT	0.00	2.51	0.012	1.01 (1.01–1.02)
Cr	0.02	1.74	0.081	1.03 (1.00–1.06)
UA	0.00	3.44	<0.001	1.01 (1.01–1.02)
K	0.83	0.14	0.890	1.12 (0.22–5.76)
Glu	0.12	0.24	0.808	1.03 (0.81–1.31)
hs-CRP	0.01	2.35	0.019	1.02 (1.01–1.04)
Lp(a)	0.00	−0.24	0.809	1.00 (0.99–1.01)
ApoA	1.08	−1.73	0.083	0.15 (0.02–1.28)
ApoB	0.83	2.13	0.033	5.86 (1.15–29.84)
cTnT	0.32	0.32	0.750	1.11 (0.59–2.09)
NTproBNP	0.00	0.37	0.714	1.00 (1.00–1.00)
WBC	0.17	0.31	0.759	1.05 (0.76–1.47)
NEUT%	0.00	0.10	0.921	1.00 (0.99–1.01)
LY%	0.04	−0.97	0.332	0.96 (0.89–1.04)
MON%	0.19	1.79	0.074	1.40 (0.97–2.02)
NEUT	0.08	3.37	<0.001	1.29 (1.11–1.49)
LYMPH	0.50	−0.63	0.528	0.73 (0.27–1.95)
MONO	1.24	1.39	0.164	5.61 (0.49–63.65)
BASO	4.38	−0.57	0.566	0.08 (0.00–434.51)
RBC	0.65	0.13	0.895	1.09 (0.31–3.89)
Hb	0.02	0.04	0.965	1.00 (0.96–1.04)
PLT	0.01	0.15	0.883	1.00 (0.99–1.01)
PT	0.22	0.11	0.913	1.02 (0.67–1.56)
INR	2.72	−0.22	0.827	0.55 (0.00–113.33)
APTT	0.01	1.75	0.080	1.01 (1.00–1.03)
FIB	0.46	−0.19	0.850	0.92 (0.38–2.24)
TT	0.01	1.26	0.209	1.01 (1.00–1.02)
D-Dimer	2.08	−0.70	0.483	0.23 (0.00–13.62)
BUN	0.15	1.06	0.288	1.17 (0.88–1.56)
Hcy	0.02	1.10	0.271	1.02 (0.98–1.06)
T3	1.65	−1.66	0.096	0.06 (0.00–1.63)
T4	0.02	−1.08	0.282	0.98 (0.95–1.01)
TSH	0.08	1.78	0.074	1.15 (0.99–1.34)
Asp	0.01	0.24	0.807	1.00 (0.99–1.01)
CK	0.00	2.08	0.038	1.01 (1.01–1.01)
CKMB	0.00	1.42	0.157	1.00 (1.00–1.01)
LDH	0.00	3.25	0.001	1.01 (1.01–1.01)
HBDH	0.00	3.08	0.002	1.01 (1.01–1.01)
LA	0.04	0.94	0.345	1.03 (0.96–1.11)
LVE	0.05	0.92	0.357	1.05 (0.95–1.16)
RA	0.06	−0.19	0.850	0.99 (0.87–1.12)
RV	0.12	0.44	0.663	1.05 (0.83–1.33)
LVEF	0.03	−3.02	0.002	0.92 (0.87–0.97)
PAP	0.05	1.24	0.215	1.07 (0.96–1.18)
TRL-C(lg)	0.91	3.76	<0.001	30.31 (5.11–179.86)

This table presents the results of univariate Cox proportional hazards regression analysis to explore the association between various clinical, laboratory, and echocardiographic factors and survival outcomes (overall survival). The dependent variable was the time-to-event outcome (all-cause death), and each independent variable was analyzed individually to evaluate its potential prognostic value.

For variables with skewed distributions [triglyceride-cholesterol ratio (TRL-C)], logarithmic transformation (lg, base 10) was performed to meet the statistical assumptions of the Cox regression model. Statistical indicators are defined as follows: S.E: Standard error of the regression coefficient, reflecting the precision of the coefficient estimate; Z: Z-statistic for testing the null hypothesis that the regression coefficient equals zero; P: Two-tailed *P* value, with *P* < 0.05 considered statistically significant; HR (95% CI): Hazard ratio with 95% confidence interval, indicating the relative risk of the study event (death) associated with a one-unit increase in the continuous independent variable. An HR > 1 suggests a risk-increasing factor, while HR < 1 indicates a protective factor.

**Table 5:** Results of multivariate COX regression.

Variables	S.E	Z	*P*	OR (95% CI)
GGT	0.01	2.88	0.004	1.02 (1.01–1.03)
LVEF	0.04	−2.31	0.021	0.92 (0.86–0.99)
TRL-C(lg)	0.98	3.44	<0.001	29.29 (4.28–200.43)

This table presents the results of multivariate Cox proportional hazards regression analysis to identify independent clinical predictors of all-cause mortality. The dependent variable was the time-to-event outcome of all-cause mortality (including follow-up time and status of all-cause death), and the independent variables included gamma-glutamyl transferase (GGT), left ventricular ejection fraction (LVEF), and log-transformed triglyceride-cholesterol ratio [TRL-C(lg)]. These variables were selected based on prior univariate Cox regression analysis (*P* < 0.05) to ensure potential prognostic relevance and statistical rationality, with adjustment for confounding factors among the variables.

TRL-C was subjected to logarithmic transformation (lg, base 10) due to its skewed distribution, which ensured compliance with the statistical assumptions of the Cox regression model. Statistical indicators are defined as follows: S.E: Standard error of the regression coefficient, reflecting the precision of the coefficient estimate; Z: Z-statistic for testing the null hypothesis that the regression coefficient equals zero; P: Two-tailed *P* value, with *P* < 0.05 considered statistically significant; HR (95% CI): Hazard ratio with 95% confidence interval, indicating the relative risk of all-cause death associated with a one-unit increase in the continuous independent variable. An HR > 1 suggests an independent risk factor for all-cause mortality, while HR < 1 denotes an independent protective factor.

**Figure 4:**
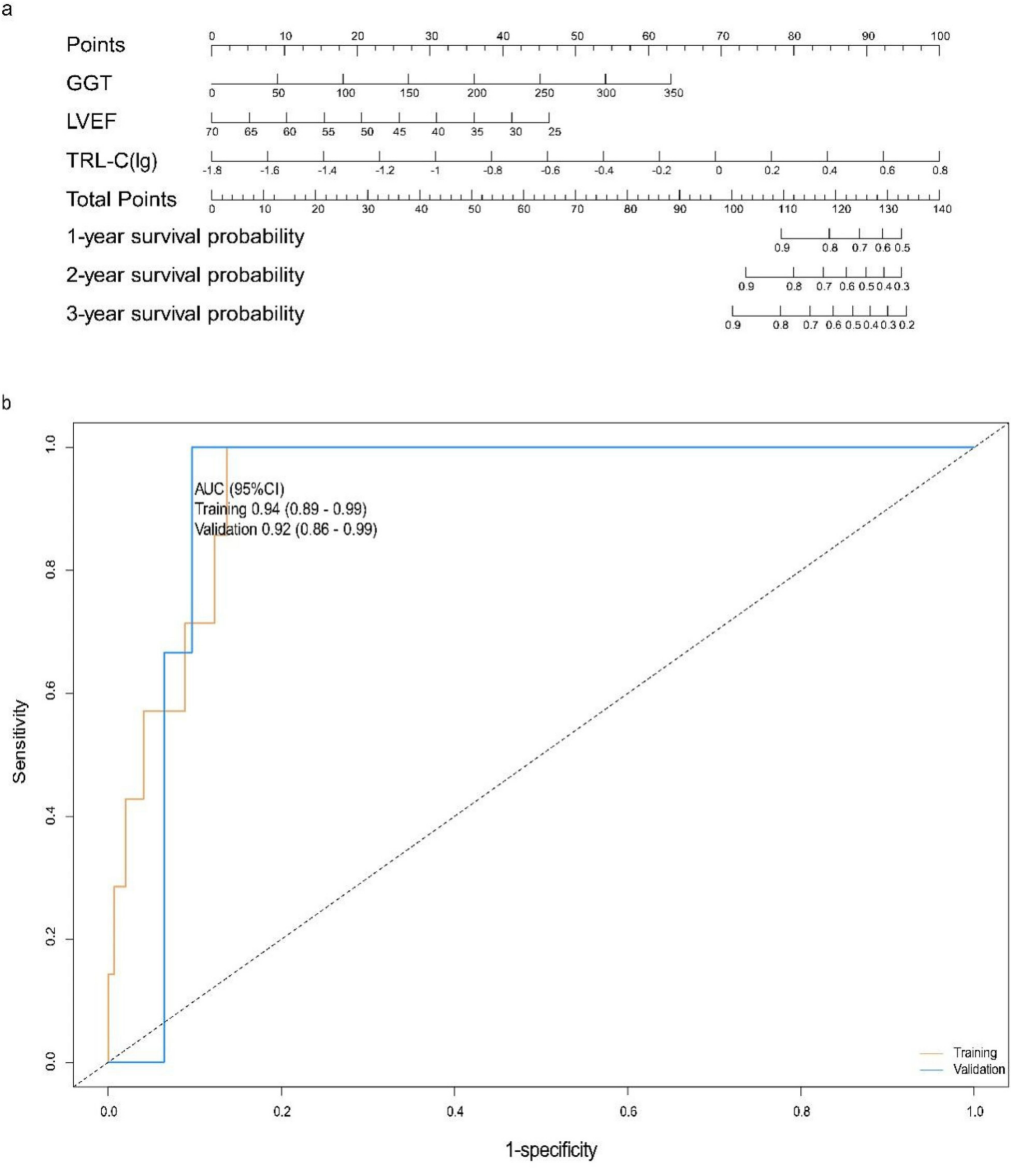
Construction and evaluation of the all-cause mortality prediction model for elderly patients with coronary heart disease. **(a)** Nomogram for predicting 1-, 2-, and 3-year survival probabilities: Clinical variables [GGT, gamma-glutamyl transferase; LVEF, left ventricular ejection fraction; TRL-C(lg), log-transformed triglyceride-rich lipoprotein cholesterol] are assigned corresponding points (top axis); the total points (sum of individual variable points) map to the predicted 1-, 2-, and 3-year survival probabilities (right axes). Each variable is scored on a scale of 0 to 100, and then the scores for each variable are summed. This total is located on the total score axis, allowing us to predict the probability of patient survival. For GGT, the scale ranges from 0 to 350. For LVEF, the scale ranges from 25 to 70. For TRL-C, the variable takes the logarithmic value, with a scale range of −1.8 to 0.8. **(b)** ROC curves for the training set and validation set. AUC, the area under the receiver-operating characteristic. ROC curves for the model in training (tan) and validation (blue) cohorts: The area under the curve (AUC, 95% confidence interval) is 0.94 (0.89–0.99) in the training cohort and 0.92 (0.86–0.99) in the validation cohort, indicating strong discriminative performance of the model in both datasets.

The partial regression coefficient (B) for TRL-C is 3.38, *P* < 0.001, which is statistically significant, with an OR value of 29.29 and a 95% confidence interval for the OR value of (4.28–200.43). TRL-C is a risk factor for all-cause mortality in elderly patients with coronary heart disease (Table 5). The partial regression coefficient (B) for GGT is 0.02, *P* = 0.004, which is statistically significant, with an OR value of 1.02 and a 95% confidence interval for the OR value of (1.01–1.03). Relatively high levels of GGT are a risk factor for all-cause mortality in elderly patients with coronary heart disease (Table 5). The partial regression coefficient (B) for LVEF is −0.08, *P* = 0.021, which is statistically significant, with an OR value of 0.92 and a 95% confidence interval for the OR value of (0.86–0.99). Relatively, LVEF is a protective factor against all-cause mortality in elderly patients with coronary heart disease (Table 5). Using the predictive factors from the training cohort multivariate COX regression, a nomogram prediction model was established, selecting three time points: one year, two years, and three years (Figure 4a). The total score can be calculated by summing the points for each variable, indicating the probability of all-cause mortality for each patient.

### 3.5 Evaluation of the all-cause mortality prediction model for elderly patients with coronary heart disease

**Figure 5:**
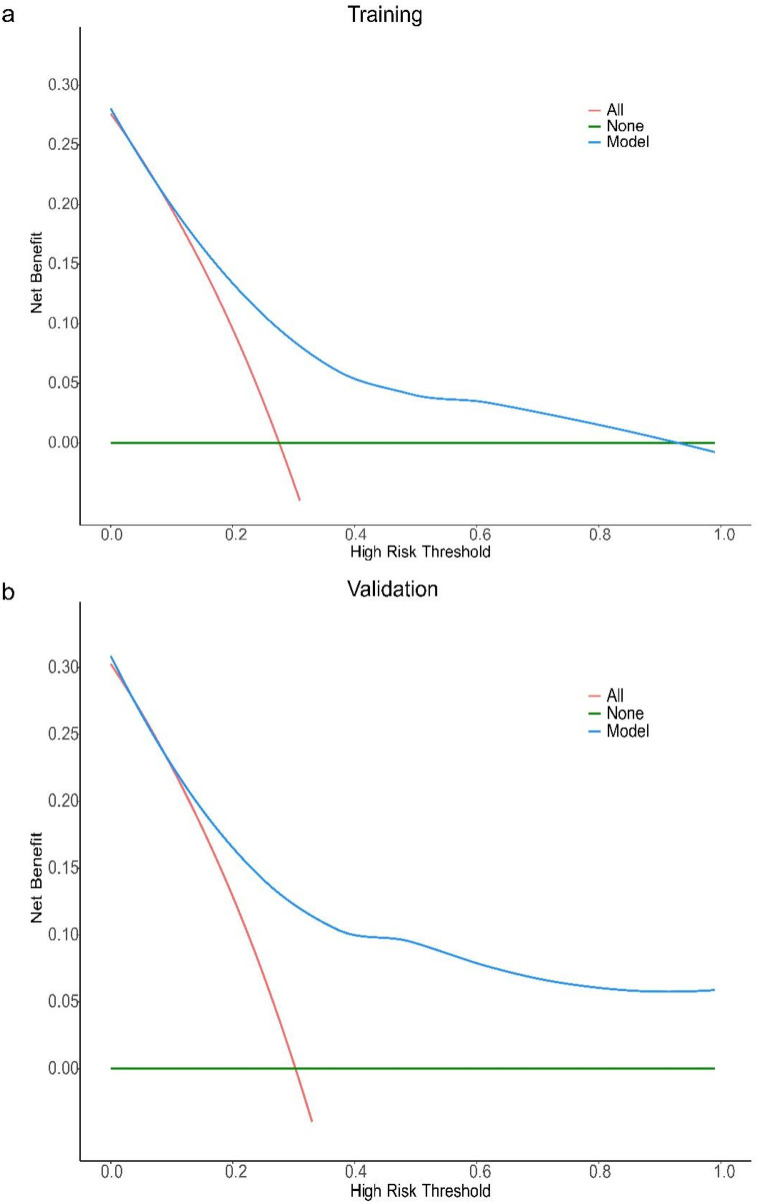
DCA curve of the all-cause mortality prediction model for elderly patients with coronary heart disease. Decision curve analysis (DCA) for the model in training **(a)** and validation **(b)** cohorts. The *x*-axis represents the high-risk threshold (probability of the event), and the *y*-axis represents the net benefit. The “Model” curve (blue) demonstrates the net benefit of using the proposed model to guide decision-making; the “All” curve (red) represents the scenario where all individuals are classified as high-risk; the “None” curve (green) represents the scenario where no individuals are classified as high-risk. In both cohorts, the proposed model (blue curve) maintains a higher net benefit across most thresholds (vs. “All” and “None”), indicating favorable clinical utility.

For the all-cause mortality prediction model for elderly patients with coronary heart disease, the ROC curve showed that the AUC for the training and validation sets was 0.94 (95% CI: 0.89–0.99) and 0.92 (95% CI: 0.86–0.99) (Figure 4b). The results of DCA show (Figures 5a,b): In both the training cohort and the validation cohort, the “Model” curve corresponding to this model consistently remains above the “All” (all determined high risk) and “None” (all not determined high risk) curves across all high-risk threshold ranges (0–1.0), indicating that the model can provide a net clinical benefit superior to “extreme strategies” by balancing true positive gains and false positive risks at any clinical decision threshold.

## 4 Discussion

Currently, coronary heart disease is a major health threat faced by the elderly population in China. Dyslipidemia is an important risk factor for coronary heart disease; in recent years, some clinical studies have demonstrated that the non-traditional lipid indicator TRL-C is closely related to the occurrence and development of atherosclerotic lesions (2). However, its pathogenicity in cardiovascular diseases has not been fully proven (1). Although evidence of the correlation between TRL-C and cardiovascular diseases is increasing, most of these studies are based on database analyses or focus on populations in Europe and America (6, 9, 13, 14). Currently, research on the correlation between TRL-C and cardiovascular diseases, especially coronary heart disease, in the Chinese population is still insufficient. Moreover, TRL-C is potentially associated with the risk of mortality in elderly patients with coronary heart disease, but existing evidence has the following limitations: triglyceride-rich lipoprotein (TRL) residual particles can increase the risk of cardiovascular events by promoting vascular endothelial inflammatory responses and accelerating the instability of atherosclerotic plaques (5). Currently, clinical studies on TRL-C mainly focus on predicting cardiovascular events in white populations, but analyses of the correlation between TRL-C and all-cause mortality in elderly patients with coronary heart disease in our population often lack specific data on mortality endpoints (15). Therefore, this study conducted a correlation analysis between TRL-C levels and the severity of coronary artery lesions in patients with coronary heart disease in China, as well as an analysis of the correlation with all-cause mortality. Unlike most previous studies conducted in Western populations, our study provides novel evidence from an elderly Chinese cohort, demonstrating the association of TRL-C with coronary lesion severity based on the SYNTAX score and with long-term outcomes. Based on the SYNTAX score, patients were divided into a “mild coronary artery lesion group” (SYNTAX 1–22) and a “moderate to severe coronary artery lesion group” (SYNTAX ≥23). Univariate analysis showed that compared to the “mild coronary artery lesion group”, the “moderate to severe coronary artery lesion group” had statistically significant differences in high-density lipoprotein cholesterol, triglycerides, activated partial thromboplastin time, thrombin time, and TRL-C. Applying these variables to a non-conditional logistic regression model for multivariate analysis, the results indicated that TRL-C is a risk factor for moderate to severe coronary artery lesions, while relatively high levels of HDL-C are protective factors. This result suggests that TRL-C has greater predictive value for the severity of coronary artery lesions in patients compared to TC or LDL-C. Model evaluation results showed that the ROC curve indicated an AUC of 0.76 (95% CI: 0.68–0.85) for the training set and 0.77 (95% CI: 0.64–0.90) for the validation set. The calibration curve also demonstrated good consistency between observed results and predicted probabilities. Additionally, DCA results indicated that the model has good clinical utility. During our 4-year follow-up, a total of 13 deaths were observed (overall mortality rate: 5.9%). Through a detailed review of medical records, we determined the causes of death: cardiac death: 8 cases (accounting for 61.5% of all deaths, 3.6% of the total cohort); non-cardiac death: 5 cases (for example, tumor, infection, etc.). Furthermore, based on follow-up results, this study constructed a prediction model for all-cause mortality in elderly patients with coronary heart disease, indicating that GGT and TRL-C are risk factors for predicting all-cause mortality in elderly patients with coronary heart disease, while a relatively high LVEF is a protective factor for predicting all-cause mortality in these patients. It is worth noting that the proportion of patients with reduced LVEF (<50%) was relatively small, which may have limited the model's ability to capture the full diagnostic impact of left ventricular dysfunction. Nevertheless, TRL-C has strong predictive value for both the severity of coronary artery lesions and all-cause mortality in elderly patients with coronary heart disease. The nomogram, together with ROC and DCA analyses, provides an intuitive and quantitative framework to assist clinicians in estimating individualized risk based on TRL-C levels. These tools can support clinical decision-making by identifying elderly patients at higher risk of severe coronary lesions or poor outcomes, thereby facilitating early intervention and optimized management strategies.

TRL-C plays an important role in the occurrence and development of atherosclerosis. TRL-C can penetrate the endothelial layer and be engulfed by macrophages, forming foam cells (16, 17). The apolipoproteins (ApoB, ApoE, ApoC-III) in TRL-C interact with proteoglycans in the subendothelium, leading to the deposition of TRL-C in the arterial wall (18). Additionally, the accumulation of TRL-C in plasma increases blood viscosity by inhibiting plasminogen activation and amplifies the coagulation cascade by promoting platelet aggregation, thrombosis, and increasing the expression of plasminogen activator inhibitor-1 (6, 19, 20). TRL-C can also upregulate the expression of endothelial cell adhesion molecules, thereby promoting the recruitment and transmigration of leukocytes and monocytes into the subendothelial space, where they accumulate and initiate inflammatory activity (21, 22). In addition, TRL-C enhances the generation of reactive oxygen species (ROS), inducing endothelial cell injury and increasing vascular permeability (23). The lipolysis of TRL releases oxidized free fatty acids, which in turn stimulate the expression of inflammatory cytokines and interleukins, amplifying vascular inflammation (24). Collectively, these processes foster the initiation and progression of atherosclerotic lesions. The atherogenic effect of TRL-C reasonably explains the findings of this study, where patients in the moderate to severe coronary artery lesion group had higher TRL-C levels, and TRL-C has good predictive value for the severity of coronary artery lesions. In clinical practice, the two models constructed in this study can assist in identifying high-risk elderly patients, providing a decision-making basis for strengthening lipid-lowering, optimizing cardiac function, and metabolic interventions.

This single-center study, based on a population from Tianjin, may involve potential selection bias, which could limit the generalizability of our findings to other regions or populations. To some extent, this population can reflect the situation of elderly patients in China; however, due to the vast territory of China, populations in different regions have varying ethnicities, lifestyles, dietary habits, and health conditions, which may lead to different lipid-related residual cardiovascular risks. Particularly, studies have shown that the obesity rate in the Tianjin population is relatively high and continues to rise (25), which may result in a higher lipid-related residual cardiovascular risk in this region. Therefore, future research should include patients from multiple regions and centers to further eliminate research biases caused by ethnicity, dietary and lifestyle habits, and health conditions.

In this study, TRL-C was calculated using a formula, with the overall median TRL-C for patients being 0.48 mmol/L, the median TRL-C for the moderate to severe coronary artery lesion group being 0.71 mmol/L, and the median TRL-C for the mild coronary artery lesion group being 0.41 mmol/L; all of which are below the reference range of “fasting level <0.8 mmol/L” provided by the 2022 Chinese Expert Consensus on Non-Traditional Lipid Indicators and Atherosclerotic Cardiovascular Disease Risk Management. This expert consensus did not provide literature references for the TRL-C reference range, which may be inferred from lipid studies in Western populations or calculated based on the normal reference ranges of TC, HDL-C, and LDL-C. This study accurately reflects the TRL-C levels in elderly patients with coronary heart disease in Tianjin, and the lower levels compared to the reference range may reflect differences in lipid levels among different ethnic groups. Based on the available information, it is difficult to determine whether the observed difference in TRL-C levels is mainly attributable to ethnic variation or methodological differences. Although ethnic diversity within China cannot fully represent global racial differences, we are currently conducting further research in Yunnan Province, where multiple ethnic minority populations live under markedly different natural climates, living environments, and dietary habits compared with the Han population in Tianjin. Such comparisons may offer insightful clues and partially help to interpret the question regarding whether the observed differences in TRL-C levels are associated with ethnic or methodological factors.

It is worth noting that the estimation of TRL-C in this study was primarily based on indirect calculation rather than direct measurement. Although this approach is practical for large-scale studies, its accuracy may vary depending on the lipid measurement methods, the formulas used to estimate LDL-C, and the levels of triglycerides. Inter-laboratory differences and the lack of standardized procedures may also contribute to measurement variability. Furthermore, The lipid profile was measured during the acute phase, when transient reductions in lipid levels may occur following myocardial infarction, potentially introducing bias in evaluating the true lipid-related risk. It should be noted that the estimation of TRL-C in this study was based on the Friedewald equation, which calculates LDL-C as TC−HDL-C−TG/5. Because the LDL-C value derived from this formula inherently includes part of the IDL-C fraction, the TRL-C calculated as TC−HDL-C−LDL-C(Friedewald) may exclude some IDL-C and thus slightly underestimate the total triglyceride-rich lipoprotein cholesterol. This underestimation is a well-recognized methodological limitation of formula-based LDL-C estimation and cannot be fully corrected without direct measurement of lipoprotein subfractions. Despite this limitation, the use of the Friedewald equation remains widely accepted in both clinical and epidemiological studies.These limitations should be carefully considered when interpreting research findings related to TRL-C.

A potential collinearity between TRL-C and HDL-C may exist because of their mathematical relationship. we conducted a rigorous multicollinearity diagnosis. The variance inflation factors (VIFs) for all variables included in the model were well below 5 (VIF for HDL-C = 1.144; VIF for TRL-C = 1.768). These results indicate that, within our dataset, the degree of collinearity among these variables has not reached a level that would substantially distort the regression coefficients. Although the multicollinearity was not statistically severe, it remains a methodological consideration worth noting.

Because of the limited number of deaths, we were unable to analyze the association between TRL-C and cardiovascular mortality. The significant link between TRL-C and all-cause mortality may be influenced by non-cardiovascular events, potentially masking its true cardiovascular impact. Further studies with larger samples and longer follow-up are warranted.

In this study, the angiographic conditions of each patient were independently calculated by two different senior interventional cardiologists from our center, which ensures the accuracy of the SYNTAX score calculations in this study. However, some scholars have pointed out that the assignment of adverse coronary features in the SYNTAX score lacks a sufficiently reliable basis, and relying solely on the SYNTAX score may not adequately reflect the severity of coronary artery lesions in patients (26). Recent studies in Asian populations have further highlighted the importance of integrating additional objective metrics for cardiovascular risk stratification. For instance, the progression of coronary artery calcium (CAC) remains low among asymptomatic individuals with baseline CAC = 0 and low-to-moderate cardiovascular risk, with a Framingham risk score below 11.1 helping identify truly low-risk individuals (27). Moreover, meta-analysis data suggest that a zero CAC score confers a “warranty period” of roughly five years, beyond which subclinical CAC progression becomes more likely, with gender-related differences in progression rates (28). These findings collectively underscore that integrating imaging modalities such as CAC assessment or intravascular techniques like OCT with angiographic scoring systems may enhance the precision of coronary lesion evaluation and patient risk stratification. In future studies, integrating OCT-based imaging findings with genetic and biomarker profiling may further improve prognostic assessment validity and provide a more comprehensive understanding of the underlying biological mechanisms. Nevertheless, this study has several limitations. It was conducted at a single center with a relatively modest sample size, which may limit the generalizability of the findings. In addition, although the nomogram exhibited good discrimination and calibration in internal validation, external validation using an independent cohort was not performed. Therefore, future multicenter studies with larger and independent populations are warranted to further confirm the robustness and applicability of our results.

## Data Availability

The original contributions presented in the study are included in the article/Supplementary Material, further inquiries can be directed to the corresponding author/s.

## Glossary

ALB: Albumin
LDL-C: Low-Density Lipoprotein Cholesterol
HDL-C: High-Density Lipoprotein Cholesterol
TG: Triglyceride
TC: Total Cholesterol
GGT: Gamma-Glutamyl Transferase
Cr: Creatinine
UA: Uric acid
K: Potassium
Glu: Glucose
hs-CRP: High-sensitivity C-reactive protein
Lp(a): Lipoprotein(a)
ApoA: Apolipoprotein A
ApoB: Apolipoprotein B
cTnT: cardiac troponin T
NTproBNP: N-terminal pro b-type Natriuretic Peptide
WBC: White Blood Cell
NEUT%: Neutrophil percentage
LY%: Lymphocyte percentage
MON%: Monocyte percentage
NEUT: Neutrophil Count
MONO: Monocyte Count
LYMPH: Lymphocyte Count
BASO: Basophilic Granulocyte Count
RBC: Red Blood Cell
Hb: Hemoglobin
PLT: Platelet Count
PT: Prothrombin Time
INR: International Normalized Ratio
APTT: Activated Partial Thromboplastin Time
FIB: Fibrinogen
TT: Thrombin Time
BUN: Blood Urea Nitrogen
Hcy: Homocysteine
T3: Triiodothyronine
T4: Tetraiodothyronine
TSH: Thyroid-Stimulating Hormone
Asp: Aspartic acid
CK: Creatine Kinase
CKMB: Creatine Kinase Isoenzyme
LDH: Lactate Dehydrogenase
HBDH: α-Hydroxybutyrate Dehydrogenase
LA: Left Atrial Diameter
LVE: Left Ventricular End-Diastolic Dimension
RA: Right Atrium Diameter
RV: Right Ventricular Diameter
LVEF: Left Ventricular Ejection Fraction
PAP: Pulmonary Arterial Pressure
TRL-C(lg): Triglyceride-Rich Lipoprotein Cholesterol

## References

[1] Navarese EP Vine D Proctor S Grzelakowska K Berti S Kubica J Independent causal effect of remnant cholesterol on atherosclerotic cardiovascular outcomes: a mendelian randomization study. Arterioscler Thromb Vasc Biol. (2023) 43(9):e373–e80. 10.1161/atvbaha.123.319297 37439258

[2] Roth GA Mensah GA Johnson CO Addolorato G Ammirati E Baddour LM Global burden of cardiovascular diseases and risk factors, 1990–2019: update from the gbd 2019 study. J Am Coll Cardiol. (2020) 76(25):2982–3021. 10.1016/j.jacc.2020.11.010 33309175 PMC7755038

[3] Zoungas S Curtis AJ McNeil JJ Tonkin AM. Treatment of dyslipidemia and cardiovascular outcomes: the journey so far–is this the End for statins? Clin Pharmacol Ther. (2014) 96(2):192–205. 10.1038/clpt.2014.86 24727468

[4] Cao YX Zhang HW Jin JL Liu HH Zhang Y Xu RX Prognostic utility of triglyceride-rich lipoprotein-related markers in patients with coronary artery disease. J Lipid Res. (2020) 61(9):1254–62. 10.1194/jlr.RA120000746 32641433 PMC7469882

[5] Xu D Xie L Cheng C Xue F Sun C. Triglyceride-rich lipoproteins and cardiovascular diseases. Front Endocrinol. (2024) 15:1409653. 10.3389/fendo.2024.1409653 PMC11176465 38883601

[6] Ginsberg HN Packard CJ Chapman MJ Borén J Aguilar-Salinas CA Averna M Triglyceride-rich lipoproteins and their remnants: metabolic insights, role in atherosclerotic cardiovascular disease, and emerging therapeutic strategies-a consensus statement from the European atherosclerosis society. Eur Heart J. (2021) 42(47):4791–806. 10.1093/eurheartj/ehab551 34472586 PMC8670783

[7] Nordestgaard BG. Triglyceride-rich lipoproteins and atherosclerotic cardiovascular disease: new insights from epidemiology, genetics, and biology. Circ Res. (2016) 118(4):547–63. 10.1161/circresaha.115.306249 26892957

[8] Jørgensen AB Frikke-Schmidt R West AS Grande P Nordestgaard BG Tybjærg-Hansen A. Genetically elevated non-fasting triglycerides and calculated remnant cholesterol as causal risk factors for myocardial infarction. Eur Heart J. (2013) 34(24):1826–33. 10.1093/eurheartj/ehs431 23248205

[9] Pradhan AD Paynter NP Everett BM Glynn RJ Amarenco P Elam M Rationale and design of the pemafibrate to reduce cardiovascular outcomes by reducing triglycerides in patients with diabetes (prominent) study. Am Heart J. (2018) 206:80–93. 10.1016/j.ahj.2018.09.011 30342298

[10] Vallejo-Vaz AJ Fayyad R Boekholdt SM Hovingh GK Kastelein JJ Melamed S Triglyceride-rich lipoprotein cholesterol and risk of cardiovascular events among patients receiving statin therapy in the tnt trial. Circulation. (2018) 138(8):770–81. 10.1161/circulationaha.117.032318 29618599

[11] Wang GN Wang Z Huo H Xu L. Machine learning differentiation of abdominal iga vasculitis without Purpura from appendicitis. Pediatr Res. (2025). 10.1038/s41390-025-04291-8 40750919

[12] Wei S Zhang H Li H Li C Shen Z Yin Y Establishment and validation of predictive model of ards in critically ill patients. J Transl Med. (2025) 23(1):64. 10.1186/s12967-024-06054-1 39806409 PMC11730794

[13] Varbo A Benn M Tybjærg-Hansen A Jørgensen AB Frikke-Schmidt R Nordestgaard BG. Remnant cholesterol as a causal risk factor for ischemic heart disease. J Am Coll Cardiol. (2013) 61(4):427–36. 10.1016/j.jacc.2012.08.1026 23265341

[14] Kaltoft M Langsted A Nordestgaard BG. Triglycerides and remnant cholesterol associated with risk of aortic valve stenosis: mendelian randomization in the copenhagen general population study. Eur Heart J. (2020) 41(24):2288–99. 10.1093/eurheartj/ehaa172 32267934

[15] Doi T Langsted A Nordestgaard BG. Elevated remnant cholesterol reclassifies risk of ischemic heart disease and myocardial infarction. J Am Coll Cardiol. (2022) 79(24):2383–97. 10.1016/j.jacc.2022.03.384 35710189 PMC8972554

[16] Jia X Rifai A Hussain M Martin A Agarwala S Virani A Highlights from studies in cardiovascular disease prevention presented at the digital 2020 European society of cardiology congress: prevention is alive and well. Curr Atheroscler Rep. (2020) 22(12):72. 10.1007/s11883-020-00895-z 33009957 PMC7532123

[17] Farnier M Zeller M Masson D Cottin Y. Triglycerides and risk of atherosclerotic cardiovascular disease: an update. Arch Cardiovasc Dis. (2021) 114(2):132–9. 10.1016/j.acvd.2020.11.006 33546998

[18] Salinas CAA Chapman MJ. Remnant lipoproteins: are they equal to or more atherogenic than ldl? Curr Opin Lipidol. (2020) 31(3):132–9. 10.1097/mol.0000000000000682 32332433

[19] Castillo-Núñez Y Morales-Villegas E Aguilar-Salinas CA. Triglyceride-rich lipoproteins: their role in atherosclerosis. Rev Invest Clin. (2022) 74(2):061–70. 10.24875/ric.21000416 34759386

[20] Gabani M Shapiro MD Toth PP. The role of triglyceride-rich lipoproteins and their remnants in atherosclerotic cardiovascular disease. Eur Cardiol. (2023) 18:e56. 10.15420/ecr.2023.16 37860700 PMC10583159

[21] Kraaijenhof JM Hovingh GK Stroes ESG Kroon J. The iterative lipid impact on inflammation in atherosclerosis. Curr Opin Lipidol. (2021) 32(5):286–92. 10.1097/mol.0000000000000779 34392272 PMC8452331

[22] Henein MY Vancheri S Longo G Vancheri F. The role of inflammation in cardiovascular disease. Int J Mol Sci. (2022) 23(21):12906. 10.3390/ijms232112906 36361701 PMC9658900

[23] Libby P. The changing landscape of atherosclerosis. Nature. (2021) 592(7855):524–33. 10.1038/s41586-021-03392-8 33883728

[24] Bornfeldt KE Linton MF Fisher EA Guyton JR. JCL roundtable: lipids and inflammation in atherosclerosis. J Clin Lipidol. (2021) 15(1):3–17. 10.1016/j.jacl.2021.01.005 33589093 PMC10009885

[25] Lili F Peng X Jing L Changkun L Wenlong Z Guohong J. Analysis of the changes in the rates of overweight, obesity and central obesity among adults in Tianjin from 2010 to 2018(in Chinese). Chin J Chron Dis Prevention Control. (2021) (011):029.

[26] Xu M Wang S Zhang Y Zhang J Ma J Shen J Residual coronary artery tree description and lesion evaluation (catlet) score, clinical variables, and their associations with outcome predictions in patients with acute myocardial infarction. Chin Med J. (2023) 136(20):2459–67. 10.1097/cm9.0000000000002640 37052135 PMC10586838

[27] Shen YW Wu YJ Hung YC Hsiao CC Chan SH Mar GY Natural course of coronary artery calcium progression in Asian population with an initial score of zero. BMC Cardiovasc Disord. (2020) 20(1):212. 10.1186/s12872-020-01498-x 32375648 PMC7204036

[28] Chen CL Wu YJ Yang SC Wu FZ. New look at the power of zero coronary artery calcium (CAC) in Asian population: a systemic review and meta-analysis. Cardiovasc Diagn Ther. (2024) 14(3):377–87. 10.21037/cdt-23-474 38975010 PMC11223936

