# Supplementary material for: Diagnostic value of triglyceride-rich lipoprotein cholesterol for coronary artery lesions and its predictive significance for long-term prognosis in elderly patients with coronary heart disease
Source: Front Cardiovasc Med. 2026 Jan 8;12:1638134. doi: 10.3389/fcvm.2025.1638134 (PMC12823998; doi:10.3389/fcvm.2025.1638134)
Supplement: Supplementary file 1 [file Image1.pdf]

a

b

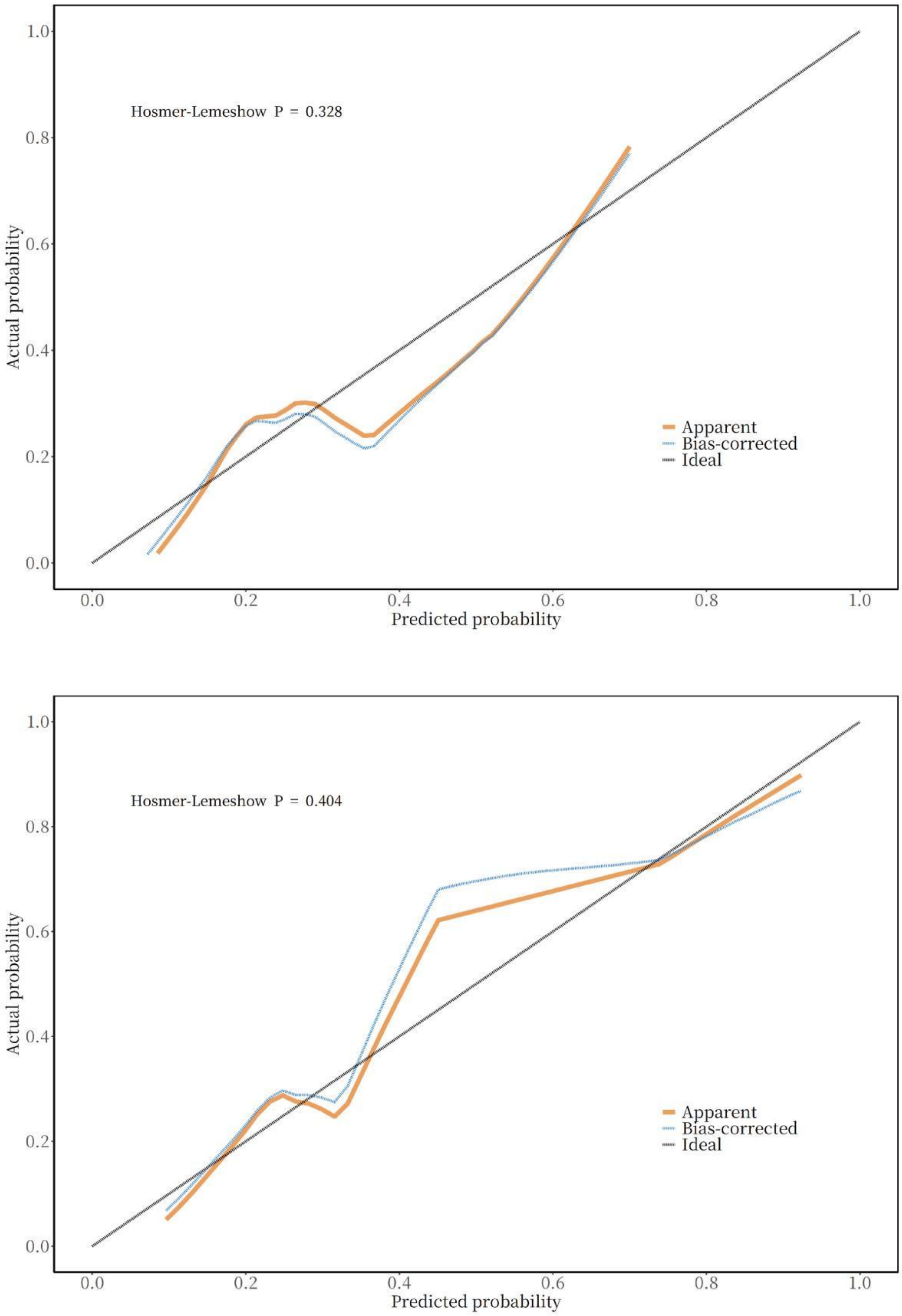

Supplementary Figure S1. The prediction model for the severity of coronary artery damage in elderly patients with coronary heart disease's J-standard curve. Figure Supplementary Figure S1 a, b. Calibration curves for the training set and validation set.
